# Dihydroartemisinin Alleviates Imiquimod-Induced Psoriasis-like Skin Lesion in Mice Involving Modulation of IL-23/Th17 Axis

**DOI:** 10.3389/fphar.2021.704481

**Published:** 2021-08-16

**Authors:** Jiang-Min Liu, Quan-Xin Jin, Manabu Fujimoto, Fang-Fang Li, Lin-Bo Jin, Ran Yu, Guang-Hai Yan, Lian-Hua Zhu, Fan-Ping Meng, Qing-Gao Zhang, Gui-Hua Jin

**Affiliations:** ^1^ Department of Immunology and Pathogenic Biology, Yanbian University Medical College, Yanji, China; ^2^ Department of Dermatology, Graduate School of Medicine, Osaka University; Laboratory of Cutaneous Immunology, Osaka University Immunology Frontier Research Center, Osaka, Japan; ^3^ Jilin Key Laboratory for Immune and Targeting Research on Common Allergic Diseases, Yanbian University Medical College, Yanji, China; ^4^ Department of Dermatology, Yanbian University Hospital, Yanji, China; ^5^ Chronic Disease Research Center, Dalian University, Dalian, China

**Keywords:** dihydroartemisinin, psoriasis, Il-23/th17 axis, cytokines, T cells

## Abstract

**Background:** Psoriasis is a T help 17 (Th17) cell-mediated chronic inflammatory skin disease. Recent studies have shown that dihydroartemisinin (DHA) can significantly reduce experimental autoimmune encephalomyelitis and rheumatoid arthritis by regulating Th17 cells.

**Objective:** To verify whether DHA can improve the symptoms of psoriasis and to further explore the possible mechanism.

**Methods:** The efficiency of DHA was preliminary detected on human keratinocytes (HaCaT) cells in psoriatic condition. Then, imiquimod-induced psoriasis-like model in BALB/c mice was established to evaluate the effects of DHA *in vivo*.

**Results:** Under the stimulation of tumor necrosis factor-α (TNF-α) and interferon-γ (IFN-γ), DHA inhibited the proliferation of HaCaT cells and significantly affected the mRNA expression levels of IFN-γ, interleukin (IL), IL-17A and IL-23. DHA treatment reduced the severity of psoriasis-like skin and resulted in less infiltration of immune cells in skin lesions. DHA restored the expression of IFN-γ, IL-17A, and IL-23 in skins, as well as a decrease of cytokines and chemokines in skin supernatant. DHA also altered the cellular composition in the spleen, which is the makeup of the T cells, dendritic cells (DCs), and macrophages. DHA recovered Th17-related profile with decreased frequency of IL-17^+^CD4^+^T cells from splenocyte of mice. Furthermore, DHA also inhibited the concentration of IL-17 from Th17 cells and the expression of Th17 cell-related transcription factors retinoid-related orphan receptor-gamma t (ROR-γt) *in vitro*. In addition, phosphorylation of signal transducer and activator of transcription-3 (STAT3) was significantly reduced in DHA treatment mice, suggesting that the IL-23/Th17 axis plays a pivotal role.

**Conclusion:** DHA inhibits the progression of psoriasis by regulating IL-23/Th17 axis and is expected to be an effective drug for the treatment of psoriasis.

## Introduction

Psoriasis is an immune-related chronic inflammatory disease characterized by excessive growth and aberrant differentiation of keratinocytes (Lowes et al., 2007). Interleukin-23/T help 17 cells (IL-23/Th17) axis was found to have a potential function in the pathogenesis of psoriasis. Dendritic cells (DCs) secrete IL-23 to bind IL-23 receptor (IL-23R) presented on Th1, Th17, and Th22 cells, which in turn act on keratinocytes (Di Cesare et al., 2009; Raychaudhuri et al., 2015), ultimately leading to excessive proliferation of the cutaneous keratinocyte to form psoriasis (Chan et al., 2006). Intradermal injection of IL-23 in mouse skin induces several features of psoriatic skin (Gauld et al., 2018). Th17 cells can initiate the proliferation, maturation, and differentiation of neutrophils to result in the inflammatory reaction, stimulate keratinocyte proliferation, and have a synergistic stimulation effect on T cell activation (Teunissen et al., 1998; Fujishima et al., 2010). Th17 cells predominantly produce IL-17, but also emerge IL-21, IL-22, interferon-γ (IFN-γ), and tumor necrosis factor (TNF) and can express C-C chemokine receptor 6 (CCR6), a chemokine (CC motif) ligand 20 (CCL20) receptor that directs IL-17 and guide cells to epithelial barrier sites (Bedoya et al., 2013).

Recent studies have indicated that the IL-23/Th17 axis plays a significant role in psoriasis. Therefore, IL-23/Th17 axis has become a potential drug treatment target. At present, the anti-IL-17 antibody antagonists are approved by the Food and Drug Administration (FDA) in the United States for clinical use, including secukinumab and ixekizumab, which are superior to TNF-α antagonists in the treatment of moderate to severe plaque psoriasis, and have good safety and tolerance (Wong et al., 2016; Dubash et al., 2019). Several IL-23 antagonists, Risankizumab (Gordon et al., 2018), guselkumab (Reich et al., 2017), and tildrakizumab (Reich et al., 2020), have completed phase III clinical trials. Although antibodies are highly desirable, the application is difficult to popularize premium prices, serious side effects, and limited corresponding sites of action (Blauvelt et al., 2015; Teraki et al., 2018; Wendling et al., 2019). Direct inhibition of IL-17 by monoclonal antibodies in patients with psoriasis or psoriatic arthritis has been shown to increase the risk of Candida infection. Similarly, latent tuberculosis infection reactivation was observed in patients treated with TNF inhibitors (Souto et al., 2014; Saunte et al., 2017).

Dihydroartemisinin (DHA) is the main active metabolite of artemisinin compounds. Some studies have found that DHA has a potent effect on the treatment of autoimmune diseases and tumors without obvious toxic and side effects. DHA inhibits the activation of Toll-like receptor 4 signal transduction pathway and the production of type I interferon and anti-ds-DNA in splenocytes from MRL/lpr lupus mice to improve the pathological damage of lupus nephritis (Huang et al., 2014). Zhao YG et al. has demonstrated the immune regulatory function of DHA in reciprocally regulating Th and regulatory T cells (Treg) generation through the modulating mammalian target of rapamycin (mTOR) pathway so as to prevent the onset of experimental autoimmune encephalomyelitis (EAE) by modestly inhibiting the proliferation of activated T cells and particularly virtually abolishing Th17 differentiation (Zhao et al., 2012). Moreover, DHA derivative DC32 inhibits the immune system imbalance and lymphocytic infiltration in synovitis of rheumatoid arthritis (RA) by restoring Treg/Th17 balance through inhibition of IL-6 (Fan et al., 2018). Nevertheless, whether DHA has an effect on psoriasis has not been reported.

Based on the above research, we hypothesized the inhibitory effect of DHA on psoriasis and preliminarily explored the possible mechanism of DHA regulating psoriasis based on the IL-23/Th17 axis for providing a potential therapeutic agent for psoriasis.

## Materials and Methods

### Cell Lines and Culture

Human keratinocytes (HaCaT cells) were purchased from FuDan IBS Cell Center (FDCC-HPN096, FDCC, Shanghai, China). Human skin fibroblasts (HSF) were established from FuHeng Cell Center (FH0186, Shanghai, China). They were maintained in DMEM medium (Gibco, United States) supplemented with 10% fetal bovine serum and 1% penicillin and streptomycin (BI, Israel). The cells were cultured at 37°C in 5% CO_2_ atmosphere.

The proliferation of keratinocytes in psoriatic conditions was induced by 10 ng/ml recombinant human TNF-α and 10 ng/ml IFN-γ to further study the effects of DHA on the expression of cytokines on HaCaT cells.

### Mice

BALB/c female mice (8–10 w) were healthy and fertile and did not display evidence of infection or disease. All mice were housed in a specific pathogen-free barrier facility and screened regularly for pathogens. All studies and procedures were approved by the Experimental Animal Ethical Committee of Yanbian University (SYXK (ji) 2020–0009).

### Psoriasis-like Model and Dihydroartemisinin Treatment

62.5 mg 5% imiquimod (IMQ, Aldara, 3M Pharmaceuticals) cream was smeared to the shaved back of mice for 5 days to induce psoriasis-like skin lesions (IMQ group). Dihydroartemisinin (DHA, National Institutes for Food and Drug Control, Beijing, China) was applied i.g. at a dose of 25 mg/kg/d (DHA-L group) and 50 mg/kg/d (DHA-H group) 3 days in advance for 8 days. As for the control group (Con. Group), they were treated similarly with control vehicle cream and phosphate-buffered saline.

The inflammation skins were graded according to the clinical Psoriasis Area and Severity Index (PASI). Erythema, scaling, and thickening were scored independently on a scale from 0 to 4: 0, none; 1, slight; 2, moderate; 3, marked; 4, very marked. The cumulative PASI score served as a measure of the severity of inflammation (scale 0–12).

### Cell Viability

HaCaT cells were placed into 96-well plates at a density of 2 × 10^4^ cells per well, then incubated overnight at 37°C in 5% CO_2_ atmosphere, and treated with different concentrations of DHA for 24 and 48 h. Cell viability was determined using MTT assays (Solarbio, China). The absorbance was measured at a wavelength of 490 nm using a microplate reader (Tecan Infinite M200 Pro, Switzerland).

### Flow Cytometry

Fluorescein isothiocyanate-, R-phycoerythrin (PE)-, peridinin chlorophyll protein (PerCP)-, PE-Cy5-, PE-Cy7-, allophycocyanin (APC)-conjugated mAbs in this study include those to CD3 (17A2), CD4 (RM4-5), CD8 (53-6.7), F4/80 (BM8), CD11c (N418) from Biolegend, IFN-γ (XMG1.2), and IL-17 (eBio17B7) from eBioscience. For intracellular staining, cells were fixed and permeabilized using Cytofix/Cytoperm kit (BD Biosciences). Dead cells were detected by LIVE/DEAD Fixable Green Dead Cell Kit (Invitrogen-Molecular Probes). Multiparameter flow cytometric analysis was performed on the FACS Verse (BD Biosciences, San Jose, Calif) and CytoFLEX (Beckman Coulter, China). Data were analyzed using FlowJo software (Ver. 8.8.7).

### Cell Isolation and Activation

Splenic T cells were purified using a CD4^+^T cell isolation kit (Miltenyi Biotec, Germany; Stemcell, Canada), CD4^+^T cells (5 × 10^4^) were activated by 10 μg/ml anti-CD3 (17A2, Biolegend) and 2 μg/ml anti-CD28 (37.51, Biolegend) for 48 or 96 h with or without 0.5 μg/ml DHA. PMA (50 ng/ml; Sigma-Aldrich) and ionomycin (500 ng/ml; Sigma-Aldrich) were included in the culture medium for the last 5 h of incubation.

### Histochemical Staining

Tissues were harvested using a disposable, sterile 6 mm punch biopsy blade (Maruho, Osaka, Japan) and assessed for tissue damage and the number of infiltrating T cells, DCs, and macrophages. Four-micrometer sections were stained using haematoxylin and eosin (H&E) to identify histologic changes of dermal tissues. To identify keratinocyte proliferation, rabbit anti-mouse Ki67 mAb (D3B5) were, respectively, used on paraffin-embedded sections by immunohistochemical (IHC) staining.

All cells were counted from the infection site in five serial skin sections. The numbers of infiltration cells were averaged in more than five power microscopic fields (HPFs, 0.07 mm^2^). Each section was examined independently by three investigators in a blinded fashion, and the meaning was used for analysis.

### Reverse Transcription Polymerase Chain Reaction

The total RNA was extracted from skin lesions and HaCaT cells using RNeasy Mini Kit (Qiagen, Hilden, Germany). Isolated RNA was reverse-transcribed to cDNA by using the M-MLV Reverse transcriptase system (Promega). cDNA was amplified with the ExTaq enzyme system. The sequences of primers were as follows: IFN-γ F: 5′-CGG​CAC​AGT​CAT​TGA​AAG​CCT​A-3′, R: 5′-GTT​GCT​GAT​GGC​CTG​GAT​TGT​C-3′; IL-4 F: 5′-ACG​GAG​ATG​GAT​GTG​CCA​AAC-3′, R: 5′-AGC​ACC​TTG​GAA​GCC​CTA​CAG​A-3′; IL-6 F: 5′-CCA​CTT​CAC​AAG​TCG​GAG​GCT​TA-3′, R: 5′-TGC​AAG​TGC​ATC​ATC​GTT​GTT​C-3′; IL-10 F: 5′-GCC​AGA​GCC​ACA​TGC​TCC​TA-3′, R: 5′-GAT​AAG​GCT​TGG​CAA​CCC​AAG​TAA-3′; IL-17A F: 5′-GAA​GGC​CCT​CAG​ACT​ACC​TCA​A-3′, R: 5′-TCA​TGT​GGT​GGT​CCA​GCT​TTC-3′; IL22 F: 5′-CCT​TCC​CCA​GTC​ACC​AGT​TG-3′, R: 5′-CTC​CAC​TCT​CTC​CAA​GCT​TTT-3′; IL23R F: 5′-GCT​CTG​AAG​TGG​AAT​TAT​GTG​C-3′, R: 5′-CTT​CTT​CTG​TCT​CTA​AAC​TCT​TCA​C-3′; IL-23 F: 5′-GAC​TCA​GCC​AAC​TCC​TCC​AGC​CAG-3′, R: 5′-TTG​GCA​CTA​AGG​GCT​CAG​TCA​GA-3′; Retinoid-related orphan receptor-gammat (ROR-γt) F: 5′-A-GCT​AGG​TGC​AGA​GCT​TCA​G-3′, R: 5′-ATT​TGT​GTT​CTC​ATG​ACT​GAG​CC-3′; β-actin F: 5′-AGG​TCA​TCA​CTA​TTG​GCA​ACG​A-3′, R:5′-CACTTCATGATGGAATTGAATGTAGTT-3′. The relative expression levels of genes were compared with β-actin.

### Western Blotting Assays

30 mg spleen tissue was minced and homogenated, and the proteins were treated and then incubated with primary antibodies against mouse STAT3 and phosphorylation of STAT3 Ab (Cell Signaling Technology) at 4°C overnight, followed by incubation with fluorescent secondary antibodies. Finally, the membranes were scanned using a Bio-Rad Gel imaging system (Bio-Rad, United States) after visualization treatment using the ECL reagent. Protein expressions were analyzed with ImageJ software.

### Enzyme-Linked Immunosorbent Assay (ELISA)

50 mg depilated back skin was homogenated in PBS and 5% FBS medium, and the supernatant was obtained by centrifugation at 5000 rpm for 15 min. IL-1β, IL-6, IL-18, and Chemokine (C-X-C motif) Ligand 1 Protein (CXCL-1) in the supernatant were determined by an ELISA kit (Mbbiology biological, China).

IL-17 secretion from purified CD4^+^T cells in the culture supernatant fluid was determined by ELISA kits (Mbbiology biological, China).

### Statistical Analysis

All data were analyzed with GraphPad Prism 9.0 (GraphPad Software, La Jolla, United States) and exhibited as the means ± SD (*n* ≥ 4 mice/group). The significance of differences was determined using the Student’s *t* test and one-way ANOVA analysis of variance followed by Dunnett’s test.

## Results

### Dihydroartemisinin Inhibited the Proliferation and Affected the Expression of Cytokines in Human Keratinocyte Cells

**FIGURE 1:**
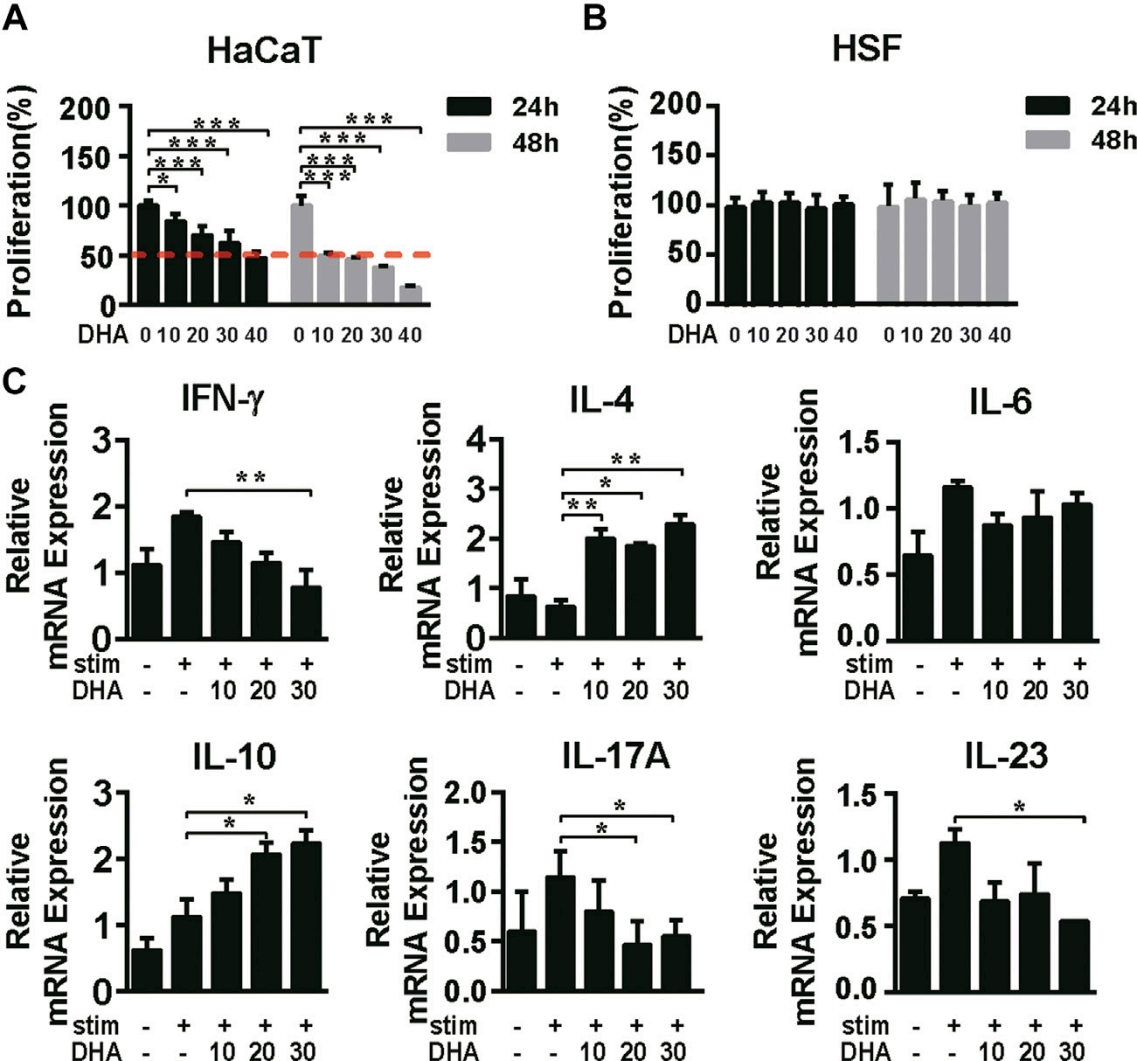
The effect of DHA on the proliferation of HaCaT cells and the expression of cytokines. **(A,B)** The cell viability of HaCaT cells and HSF cells was detected by MTT assay being treated with different concentrations of DHA for 24 and 48 h (*p = 0.0318, ***p < 0.001). **(C)** After treatment on HaCaT cells with 0, 10, 20, 30 μg/ml DHA for 24 h with the stimulation of IFN-γ (10 ng/ml) and TNF-α (10 ng/ml), the mRNA expression of inflammation-related cytokines IFN-γ, IL-4, IL-6, IL-10, IL-17A, and IL-23 was detected by RT-PCR (IFN-γ, **p = 0.0094; IL-4, *p = 0.0292, **p < 0.05; IL-6, *NS.* Not significant; IL-10, *p < 0.05; IL-17A, *p < 0.05; IL-23, *p = 0.0335). The significant difference was compared relatively to the DHA untreated group. Statistical analysis was carried out using One-way ANOVA analysis of variance followed by Dunnett’s test (*n* = 5–7).

First, we clarified the inhibitory effect of DHA on keratinocytes and cytokines associated with inflammation. The effects of DHA on the proliferation of HaCaT cells and HSF cells were initially detected. MTT results showed that DHA inhibited the proliferation of HaCaT cells (*p* < 0.05); the IC50 values of 24 and 48 h were, respectively, 32.51 μg/ml and 8.23 μg/ml. Moreover, DHA did not affect the proliferation of human skin fibroblasts HSF cells (Figures 1A,B). The psoriatic condition of HaCaT cells is mainly induced by the TNF-α/IFN-γ-stimuli. The results showed that mRNA expression of IFN-γ, IL-17A, and IL-23 was significantly decreased, while IL-4 and IL-10 were accelerated in the DHA-treated group. In contrast, DHA treatment did not change IL-6 mRNA expression levels (Figure 1C). Thus, DHA adjusted inflammation-related cytokines expression in HaCaT cells.

### Dihydroartemisinin Ameliorated Psoriasis-like Skin Lesions

**FIGURE 2:**
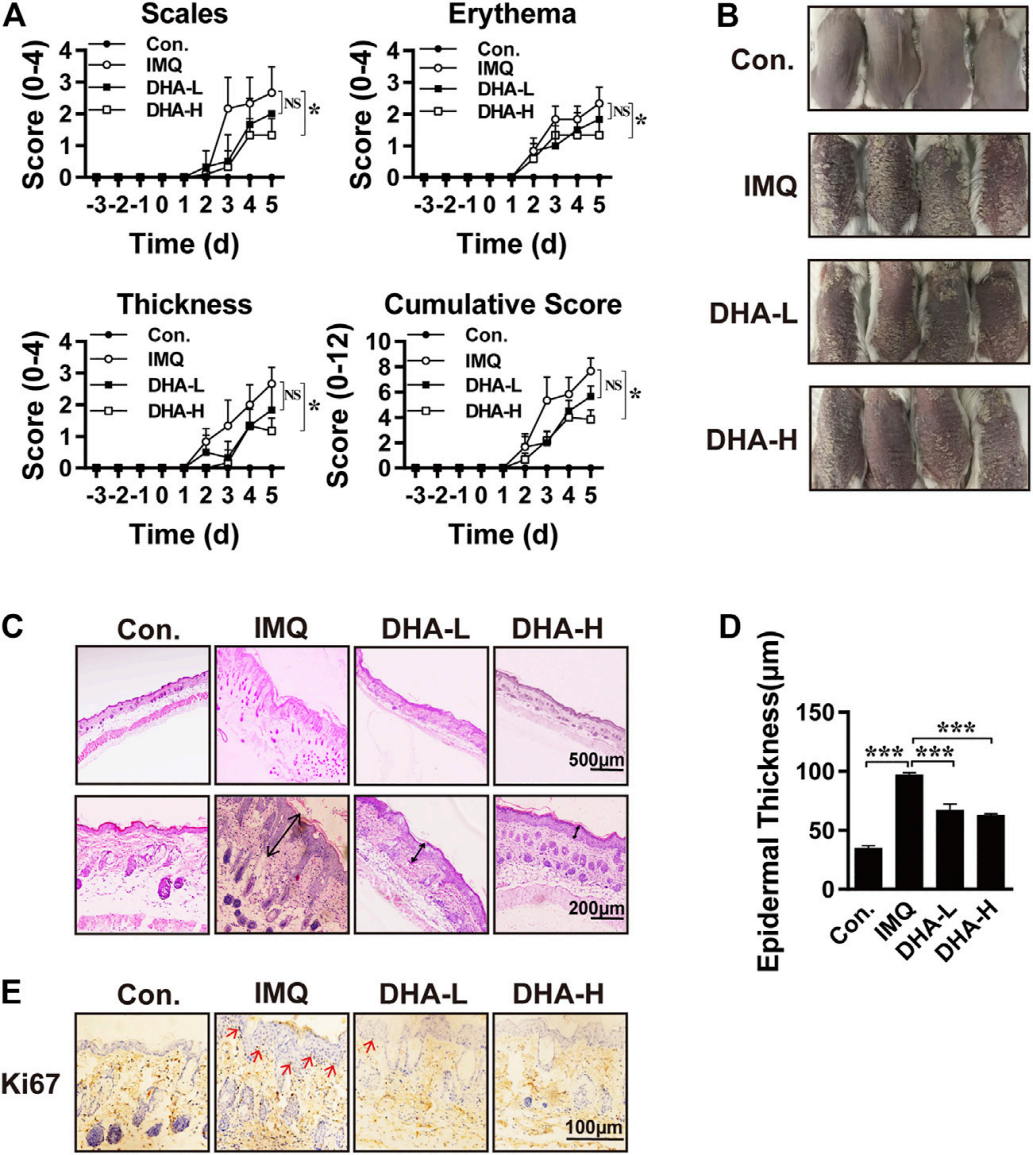
DHA treatment attenuated psoriasis severity. **(A)** These selected mice were classified into four groups as described above, clinical indicators scales, erythema, and skin thickness for PASI clinical evaluation to assess the severity of psoriasis and the efficacy of DHA (*p < 0.05, *NS.* Not significant). **(B)** Representative macroscopic skin lesion symptoms in each group. **(C)** H&E staining was performed to observe the pathological changes in mice's skin. Original magnifications, top ×40; bottom × 200. **(D)** Epidermal thickness was measured after DHA treatment in the psoriasis model (***p < 0.001). **(E)** Keratinocyte proliferation was analyzed by detecting the expression of Ki67. Original magnifications, ×400. Statistical analysis was carried out using One-way ANOVA analysis of variance followed by Dunnett’s test (*n* = 5–7).

To investigate the therapeutic effect of DHA *in vivo*, IMQ-induced psoriasis-like models were established. The symptoms of erythema, scales, and skin thickness were evidently mitigated in DHA-treated mice (Figures 2A,B). Histopathological analysis revealed that keratinization, the protrusion of the epidermis, the dilatation and congestion of capillaries, and cellular infiltration were markedly enhanced in psoriasis mice compared with wild-type mice. When compared with IMQ mice, mice receiving treatment with DHA showed marked recovery pathological changes (Figures 2C,D). Moreover, the proliferation of keratinocytes was monitored by IHC staining. As shown in Figure 2E, DHA treatment significantly reduced Ki67 positive cells compared with that in IMQ mice epidermis. Overall, these data suggested that DHA suppresses augmented psoriasis-like skin reactions.

### Dihydroartemisinin Treatment Resulted in Decreased Accumulation of Immune Cells in Skin Lesions

**FIGURE 3:**
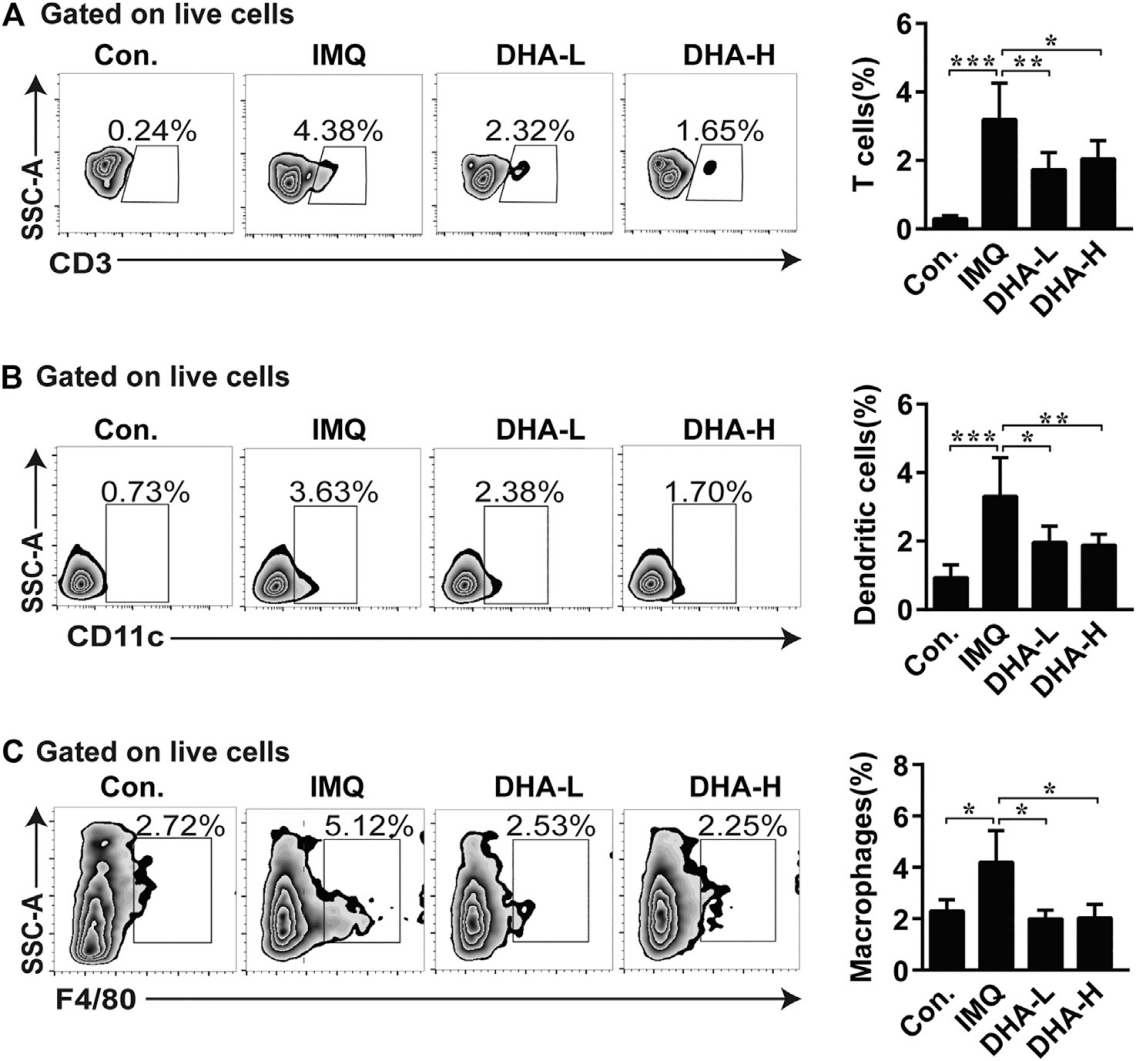
DHA treatment decreased immune cells infiltration into skins. **(A**–**C)** Percentages of T cells, macrophages, and DCs among total live cells were determined by flow cytometry (T cells, *p = 0.0328, **p = 0.0074, ***p < 0.001; macrophages cells, *p < 0.05; DCs, *p = 0.0141, **p = 0.0097, ***p < 0.001). Statistical analysis was carried out using One-way ANOVA analysis of variance followed by Dunnett’s test (*n* = 4–6).

Consistent with the report (Van der Fits et al., 2009), in the H&E sections of IMQ-treated back skin, abundant infiltrates of mononuclear cells were observed. In order to further confirm that DHA treatment can inhibit the infiltration of inflammatory cells, flow cytometry was applied. DHA-treated mice exhibited a significant decrease in T cells, macrophages, and DCs infiltration in skins compared to IMQ mice (Figure 3). The histopathological analysis also revealed that the degree of edema and cellular infiltration was markedly improved in DHA-treated mice compared with IMQ administration mice (Supplementary Figure 1). These results showed that DHA administration results in effects on the composition of the inflammatory cell.

### Dihydroartemisinin Treatment Normalized Levels of Interleukin-23/T Help 17 Cells Axis-Related Cytokines and Inflammation-Related Cytokines and Chemokines in Skins

**FIGURE 4:**
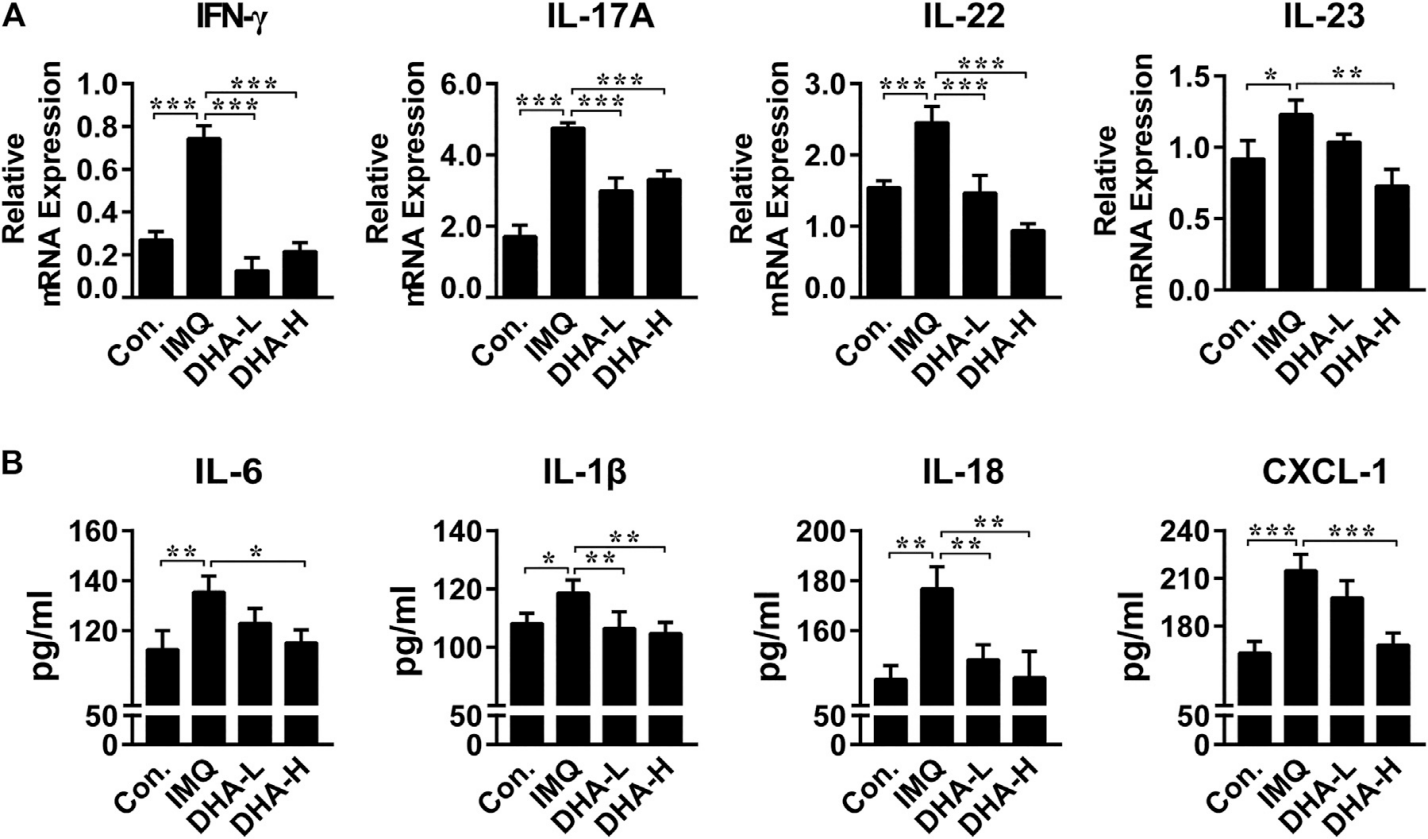
DHA affected cytokines expression in psoriasis mice skin lesions. **(A)** IFN-γ, IL-17A, IL-22 and IL-23 mRNA levels in skin sections (30 mg) were analyzed by RT-PCR (IFN-γ, ***p < 0.001; IL-17A, ***p < 0.001; IL-22, ***p < 0.001; IL-23, *p = 0.017, **p = 0.0010). **(B)** IL-6, IL-18, IL-1β and CXCL-1 secretion in the supernatant from skins (50 mg) were measured by ELISA (IL-6, *p = 0.011, **p = 0.0056; IL-18, **p < 0.01; IL-1β, *p = 0.0295, **p < 0.01; CXCL-1, ***p < 0.001). Statistical analysis was carried out using One-way ANOVA analysis of variance followed by Dunnett’s test (*n* = 4–6).

The critical role of the IL-23/Th17 axis in the development of psoriasis has been demonstrated. To further explore the effect of DHA on the IL-23/Th17 axis, we first determined mRNA expression levels of cytokines in each group. mRNA expression of IFN-γ was significantly decreased in the DHA administration group compared with the IMQ group. Likewise, IL-23/Th17 axis-related cytokines IL-17A, IL-22, and IL-23 were dramatically dropped in the DHA-treated groups (Figure 4A). Furthermore, we assessed expressions of inflammation-related cytokines and chemokines such as IL-1β, IL-6, IL-18, and CXCL-1 in skin tissues 6 days after DHA treatment, and the results showed that DHA significantly decreased expression of them (Figure 4B). Thus, DHA inhibited the expression of cytokines and chemokines in psoriasis-like skin damage.

### Dihydroartemisinin Treatment Affected the Immune Cellular Composition in Spleens

**FIGURE 5:**
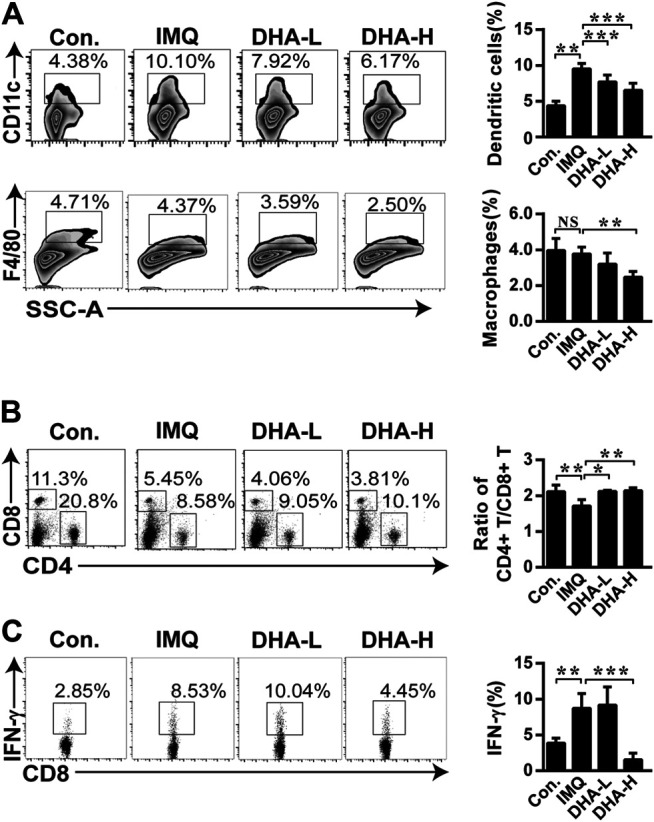
DHA treatment altered immune cellular composition in spleens. **(A)** The frequencies of DCs, and the frequencies of macrophages. **(B)** Ratio of CD4^+^/CD8^+^ T cells was analyzed by flow cytometry. **(C)** Splenocytes were stimulated by PMA and ionomycin for 4 h in the presence of brefeldin A, then frequency of IFN-γ^+^CD8^+^T cells was determined by flow cytometry (DCs, **p = 0.0027, ***p < 0.001; macrophages, **p = 0.0011; Ratio of CD4^+^/CD8^+^ T cells, *p = 0.0117, **p < 0.01; IFN-γ^+^CD8^+^T cells, **p = 0.0056, ***p < 0.001). Statistical analysis was carried out using One-way ANOVA analysis of variance followed by Dunnett’s test (*n* = 4–6).

The observed trends in spleens were also verified by flow cytometry. When compared with the control group, the frequency of DCs and macrophages was higher in the IMQ group. DHA treatment distinctly decreased the frequency of DCs and macrophages with a dose-dependent effect (Figure 5A). As shown in Figure 5B, the CD4^+^/CD8^+^T cells ratio was significantly reversed in the IMQ mice, which was recovered after DHA treatment. Furthermore, the percentage of IFN-γ^+^CD8^+^T cells significantly decreased in high-dose DHA-treated mice than that of IMQ mice (Figure 5C). Therefore, DHA treatment decreased inflammatory infiltration in the spleen of psoriasis mice.

### Dihydroartemisinin Relieved Psoriasis-like Inflammation Dependent on Interleukin-23/T Help 17 cells Axis

**FIGURE 6:**
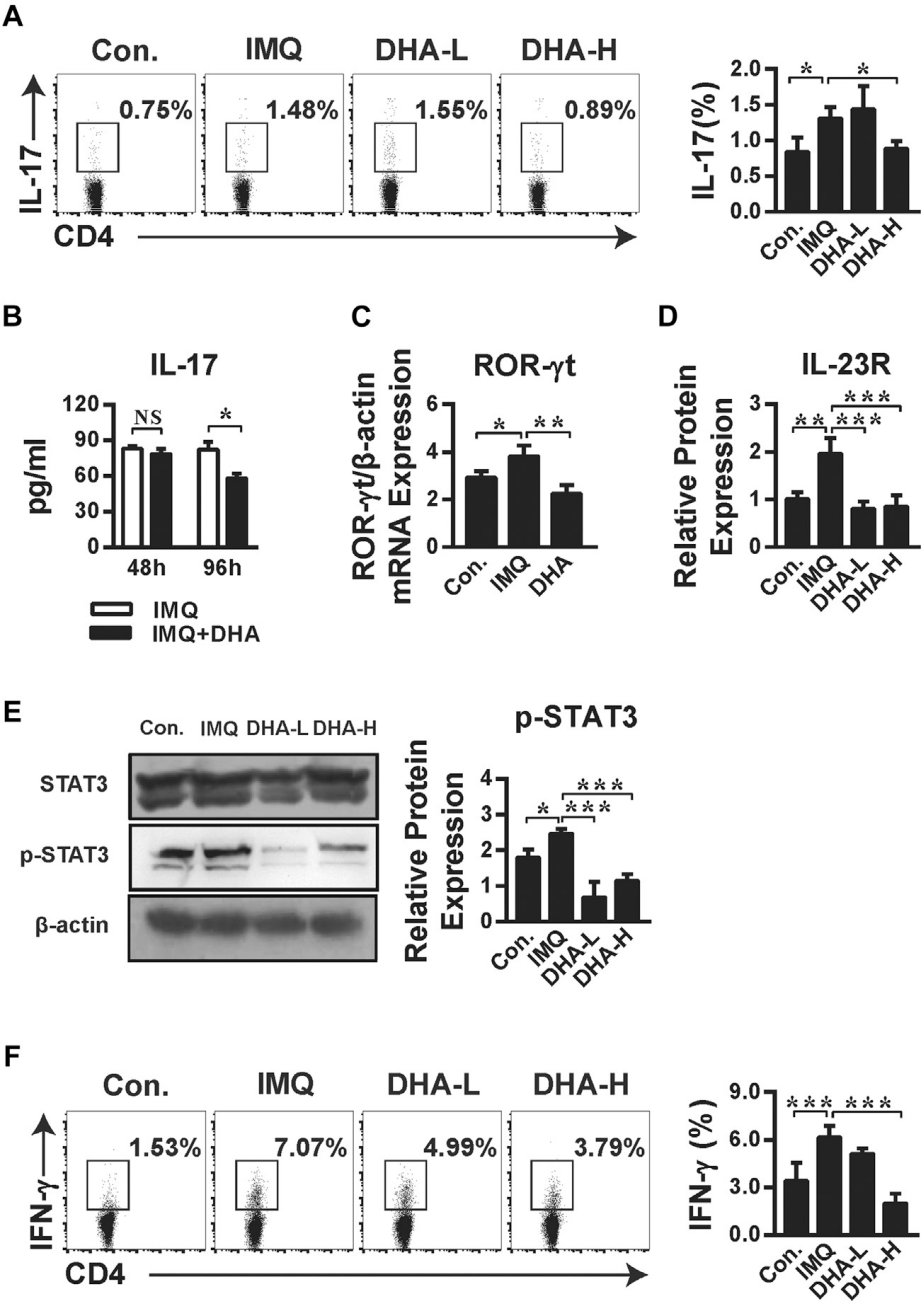
DHA restrained psoriasis dependent on IL-23/Th17 axis. **(A)** The frequency of IL-17^+^CD4^+^T cells was analyzed by flow cytometry (*p < 0.05). **(B,C)** Splenic single cell suspensions were stimulated as described in the Materials and Methods. **(B)** The concentration of IL-17 from purified CD4^+^ T cells in the culture supernatant was measured by ELISA. Statistical analysis was carried out using unpaired *t*-test (*p = 0.0304, NS. Not significant, *n* = 5–6). **(C)** ROR-γt mRNA level from Th17 cells was detected by RT-PCR (*p = 0.0448, **p = 0.0038). **(D)** IL-23R mRNA levels in skin sections (30 mg) were analyzed by RT-PCR (**p = 0.0026, ***p < 0.001). **(E)** STAT3 phosphorylation in splenocytes from control, IMQ-, and DHA-treated mice was assessed by western blotting (*p = 0.0396, ***p < 0.001). **(F)** IFN-γ production by CD4^+^T cells from spleens was measured by flow cytometric analysis (***p < 0.001). Statistical analysis was carried out using One-way ANOVA analysis of variance followed by Dunnett’s test (A, C–F; *n* = 4–6).

It has been confirmed that DHA regulated Th17 cells generation to prevent the onset of EAE (Zhang et al., 2016). To evaluate whether DHA treatment influences the Th17 cells in psoriasis, the percentage of Th17 cells and expression of differentiation-related transcription factors were detected. Indeed, DHA treatment significantly reduced the frequency of IL-17^+^CD4^+^T cells (Figure 6A). To evaluate Th17 cytokine production in spleens, CD4^+^T cells were stimulated with anti-CD3 and anti-CD28 for 48 or 96 h *in vitro*, and the expression of IL-17 was performed. IL-17 production from Th17 cells was significantly inhibited by DHA treatment after 96 h. Additionally, ROR-γt, the Th17 cell-specific nuclear transcription factor, was also reduced after DHA treatment (Figures 6B,C). Furthermore, DHA also inhibited the mRNA expression of the IL-23 receptor compared with the IMQ group. (Figure 6D). STAT3 has emerged as an important transcription factor of keratinocytes to regulate cytokine expression and Th17 cell differentiation. Phosphorylation of STAT3 was significantly decreased after DHA administration (Figure 6E). These results showed that DHA affects the IL-23/Th17 axis composed of IL-23, IL-17, STAT3, and Th17 cells. Moreover, as shown in Figure 6F, the number of IFN-γ^+^CD4^+^T cells dropped after DHA treatment, indicating that Th1 cells were also affected by DHA, suggesting that the process of DHA alleviating psoriasis likely counted on Th1/Th17 polarization, which could turn into the subsequent target.

## Discussion

In this study, we demonstrated that DHA treatment significantly inhibited the proliferation and cytokines expression of HaCaT cells. Moreover, DHA treatment significantly reduced psoriasis severity as determined by both skin score and histopathology. DHA restored IL-23/Th17 axis-related cytokine and chemokine expression and recovered polarization of Th17 cells. Therefore, the study demonstrated that DHA suppresses psoriasis-like skin lesions by regulating IL-23/Th17 axis.

The IL-23/Th17 axis is considered to play a major role in psoriasis (Gauld et al., 2019). Activated DCs and macrophages are the main source of IL-23 in psoriasis (Singh et al., 2016; Huang et al., 2018). Injection of IL-23 into the skin can induce dermal acanthosis, neutrophil aggregation, and infiltration of IL-17^+^T cells. Moreover, IL-23 inhibitors can significantly reduce the expression of IL-17 and IL-22 in psoriatic lesions (Krueger et al., 2016; Puig, 2017). In this article, we show that DC_S_ and macrophages were largely relieved after DHA treatment in skin lesions (Figure 3). In the meantime, treatment with DHA also markedly decreased DCs and macrophages infiltration in the spleen (Figure 5). Thus, DHA affects DCs and macrophages activation in psoriasis, and DCs were the main source of myeloid cell-derived IL-23.

IL-23 can induce the expression of IL-17 and assist the proliferation and survival of differentiated Th17 cells. In the absence of the IL-23 signaling pathway, Th17 cells stagnated in the early activation stage and could not regulate the encephalitis-inducing effect of EAE (Zheng et al., 2007). About 1/3 of patients with psoriasis develop psoriatic arthritis, while the number of Th17 cells in synovial fluid and synovial tissue is significantly increased, and the expression of IL-17 is also significantly increased, which demonstrates that there is a significant correlation between the level of IL-17 and joint injury. Multiple studies have demonstrated an increased expression of IL-17A and IL-17F in psoriatic skin in contrast to the nonlesion psoriatic skin, and the upregulation of IL-17A illustrated a positive association with disease severity (Lowes et al., 2008; Johansen et al., 2009). IL-17A gene could induce the proliferation of IL-17RA^+^CD11b^+^Gr1^low^ osteoclast precursors, and the biomarkers of bone resorption also increased by transferring the IL-17A gene into the CIA model, which indicated that IL-17A could induce pathological bone resorption (a crux feature of psoriatic arthritis) through direct activation of osteoclast precursors (Marinoni et al., 2014; Adamopoulos et al., 2015; Sakkas and Bogdanos, 2017). Vitamin D has been applied in the treatment of moderately severe psoriasis because DCs treated with vitamin D have lower expression of MHC-II and costimulatory molecules CD80, CD86, and CD40L, which can reduce the production of IL-12 while secreting IL-10 and ultimately inhibit the proliferation of T cells and the secretion of IFN-γ and IL-17 (Hau et al., 2018).

DHA is the main active metabolite of artemisinin, which can significantly inhibit the release of proinflammatory cytokines (IL-6, IL-1β, IL-10, TNF-α, and so on) and affect T cell differentiation, which has a favorable application prospect in anti-inflammation (Yan et al., 2019), anticancer (Dong et al., 2019), and so on (Jagannathan et al., 2016). The natural plant antimicrobial solution could significantly reduce the gene expression and inflammatory cytokines production of macrophage-derived chemokine (MDC), IL-8, and IL-6 in TNF-α/IFN-γ-induced HaCaT cells (Dou et al., 2017). Artemisinin can improve the symptoms of experimental autoimmune myasthenia gravis by regulating the balance of Th1/Th17 and influencing the function of Treg (Chen et al., 2018). DHA can similarly inhibit the secretion of TNF-α in BXSB mice and improve the pathological damage of lupus nephritis (Li et al., 2006). DHA treatment was shown to induce a decrease in IL-23, IFN-γ, and IL-17 production *in vitro* and *in vivo* (Figures 1, 4). Moreover, increased CD3^+^T cells and IFN-γ^+^CD8^+^T cells population in control groups were inhibited by DHA treatment (Figures 3, 5).

During the differentiation of Th17 cells, the signal of cytokines is transduced through STAT3, thus initiating the specific transcription program of Th17 cells. STAT3 can directly bind to IL-17A and IL-21 loci and regulate the expression of ROR-γt and IL-23R. Studies have confirmed that heterozygous STAT3 gene mutation can reduce the utilization rate of functional STAT3 dimer by 25% and consequently reduce the expression of ROR-γt by four times. The latter is a specific nuclear transcription factor necessary for Th17 cell differentiation. ROR-γt^−/−^ mice do not have Th17 cells, so leading to the immune diseases abatement (Ivanov et al., 2006; Yang et al., 2015). In the current study, the expression of phosphorylation of STAT3 was significantly inhibited by DHA administration, paralleled by a significant decrease in the expression of ROR-γt and IL-17 production from Th17 cells (Figure 6). Moreover, the IL-17^+^CD4^+^T cells population in psoriasis groups was significantly inhibited by DHA administration (Figure 6). Thus, our data showed that DHA effectively attenuates psoriasis-like skin lesions. DHA may partially regulate the IL-23/Th17 axis and polarization of Th17 cells in the psoriasis-like model. Consequently, DHA is expected to become a drug for the treatment of psoriasis in humans.

## Data Availability

The original contributions presented in the study are included in the article/Supplementary Material. Further inquiries can be directed to the corresponding author.

## Abbreviations

CCR6, CCR6 C-C chemokine receptor 6; CXCL-1, chemokine (C-X-C motif) Ligand 1 Protein; DCs, dendritic cells; HaCaT, human keratinocytes; HSF, Human skin fibroblasts; IFN-γ, interferon-γ; IL-, interleukin-; IL-23R, IL-23 receptor; mTOR, mammalian target of rapamycin; ROR-γt, retinoid-related orphan receptor-gamma t; STAT3, signal transducer and activator of transcription 3; Th17, T help 17 cells; TNF, tumor necrosis factor; Treg, regulatory T cells.
