# Supplementary figures and images for: Dihydroartemisinin Alleviates Imiquimod-Induced Psoriasis-like Skin Lesion in Mice Involving Modulation of IL-23/Th17 Axis
Source: Front Pharmacol. 2021 Aug 16;12:704481. doi: 10.3389/fphar.2021.704481 (PMC8415163; doi:10.3389/fphar.2021.704481)

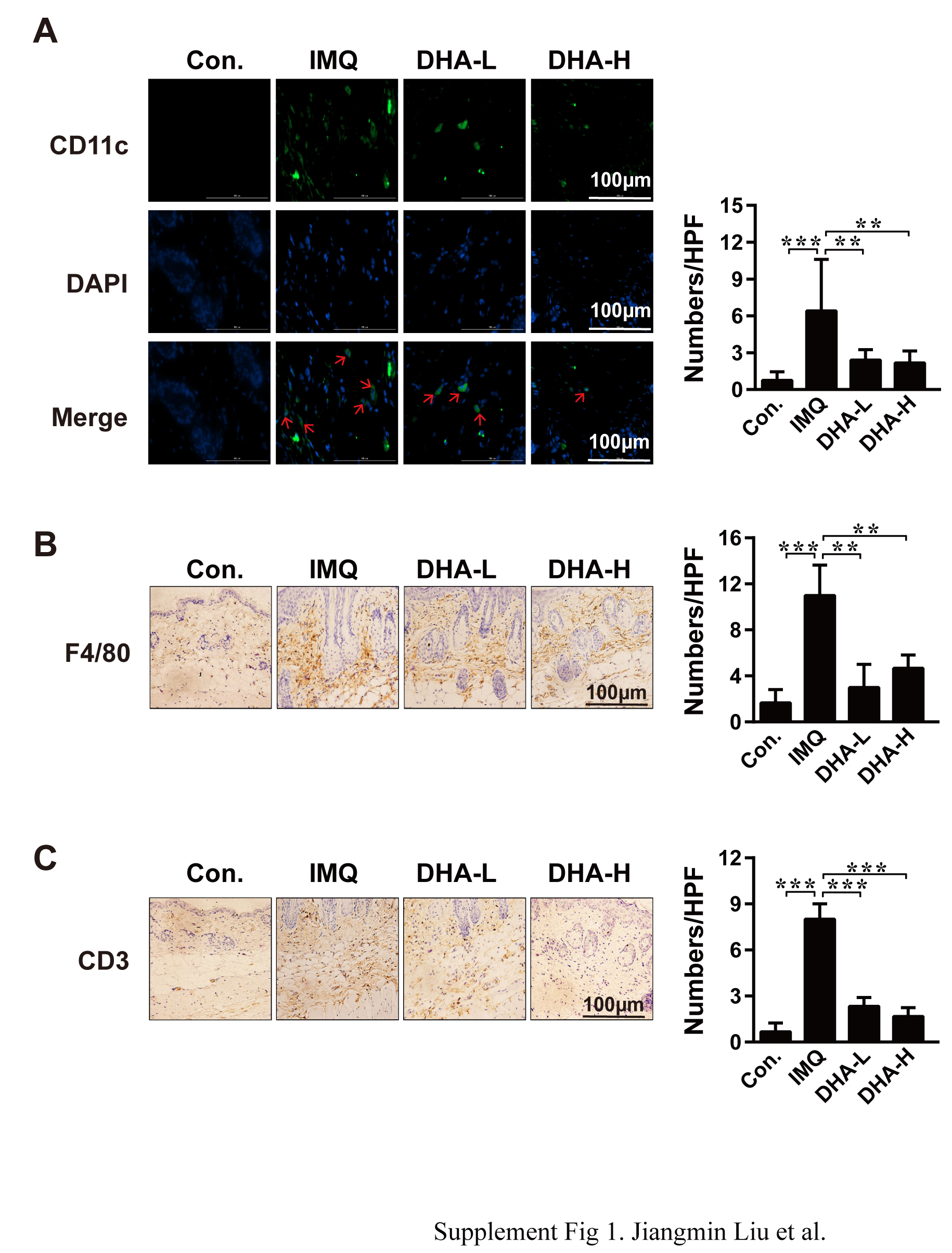

Supplement: Supplementary file 1 [file Image1.tif]
